# Pirfenidone Reduces Epithelial–Mesenchymal Transition and Spheroid Formation in Breast Carcinoma through Targeting Cancer-Associated Fibroblasts (CAFs)

**DOI:** 10.3390/cancers13205118

**Published:** 2021-10-13

**Authors:** Hamidreza Aboulkheyr Es, Thomas R Cox, Ehsan Sarafraz-Yazdi, Jean Paul Thiery, Majid Ebrahimi Warkiani

**Affiliations:** 1 School of Biomedical Engineering, University of Technology Sydney, Sydney, NSW 2007, Australia; 13279690@student.uts.edu.au; 2 The Kinghorn Cancer Centre, Garvan Institute of Medical Research, Sydney, NSW 2010, Australia; t.cox@garvan.org.au; 3 NomoCan Pharmaceuticals, New York Blood Center, New York, NY 10065, USA; ehsan.yazdi@nomocan.com; 4 Comprehensive Cancer Center, Institute Gustave Roussy, 94805 Villejuif, France; tjp@gzlb.ac.cn; 5 Guangzhou Laboratory, Guangzhou 510000, China; 6 Center of Biomedical Engineering, Sechenov University, Moscow 119991, Russia; 7 Shemyakin-Ovchinnikov Institute of Bioorganic Chemistry, Russian Academy of Sciences, Moscow 117997, Russia

**Keywords:** tumor microenvironment (TME), cancer-associated fibroblasts (CAFs), epithelial–mesenchymal transition (EMT), pirfenidone (PFD), 3D microfluidic device

## Abstract

**Simple Summary:**

Cancer-associated fibroblasts (CAFs) stimulate phenotypic transformation and acquisition of stemness in carcinoma cells. Targeting CAF-derived cytokines may suppress initiation of these events. This study aimed to show the inhibitory effects of pirfenidone on phenotypic transformation and stemness of cancer cells. To this end, we leverage the use of a 3D microfluidic device to analyze carcinoma progression phenotypes. We found that pirfenidone decreased tumor spheroid formation and epithelial–mesenchymal transition (EMT) the inhibition of cytokine production by CAFs. In the microfluidic model, we demonstrate that pirfenidone significantly inhibits the migration of carcinoma cells and CAFs. This study highlights the potential application of pirfenidone in suppressing invasion and potentially metastasis in breast cancer which can be further investigated in vivo.

**Abstract:**

The aim of this study was to assess the effects of pirfenidone (PFD) on promoting epithelial–mesenchymal-transition (EMT) and stemness features in breast carcinoma cells through targeting cancer-associated-fibroblasts (CAFs). Using The Cancer Genome Atlas (TCGA) database, we analyzed the association between stromal index, EMT, and stemness-related genes across 1084 breast cancer patients, identifying positive correlation between YAP1, EMT, and stemness genes in samples with a high-stromal index. We monitored carcinoma cell invasion and spheroid formation co-cultured with CAFs in a 3D microfluidic device, followed by exposing carcinoma cells, spheroids, and CAFs with PFD. We depicted a positive association between the high-stromal index and the expression of EMT and stemness genes. High YAP1 expression in samples correlated with more advanced EMT status and stromal index. Additionally, we found that CAFs promoted spheroid formation and induced the expression of YAP1, VIM, and CD44 in spheroids. Treatment with PFD reduced carcinoma cell migration and decreased the expression of these genes at the protein level. The cytokine profiling showed significant depletion of various EMT- and stemness-regulated cytokines, particularly IL8, CCL17, and TNF-beta. These data highlight the potential application of PFD on inhibiting EMT and stemness in carcinoma cells through the targeting of critical cytokines.

## 1. Introduction

The cellular components of the tumor microenvironment (TME) in promoting invasiveness and stemness features of carcinoma cells are well characterized [1,2]. Among them, cancer-associated fibroblast (CAFs), a major component of TME, play a key role in the regulation of tumor progression and metastasis, as well as the acquisition of stemness in carcinoma cells through the induction of various processes, including epithelial-to-mesenchymal transition (EMT) [3,4]. CAFs modulate various key factors within the TME and rewire the TME toward an aggressive ecosystem. This is achieved by the secretion of various cancer-promoting chemokines and cytokines, which activate tumor growth, and trigger cancer invasion and immune escape [5,6,7]. Besides, CAFs remodel the extracellular matrix leading to an increased stiffness which modify the phenotype in carcinoma cells [8,9]. Various studies highlighted the role of CAFs-induced Yes-activated protein (YAP1), a major regulator of cell plasticity, stemness, drug resistance, and metastasis in carcinoma cells [9,10]. Given the crucial role of CAFs within the TME, targeting these cells might be a promising therapeutic approach to reduce the invasiveness and stemness of carcinoma cells regulated by YAP1 [11]. The pirfenidone (PFD) is a well-known therapeutic agent used for the treatment of idiopathic pulmonary fibrosis (IPF). The PFD target activated fibroblasts and secretory cytokines. Numerous recent studies demonstrated the therapeutic potential of PFD as a combination treatment modality with chemotherapy, targeted therapy, and immunotherapy in various cancers through the depletion of cytokines [12,13,14,15].

Currently, the complexity of TME is commonly modelled using advanced 3D models, including tumor organoids, co-culture aggregates [16], or animal models using patient-derived xenografts [17,18]. However, the lack of real-time monitoring and controlling various variables of the microenvironment regulation in the study of TME is challenging in these model systems. These limitations prompted the development of a cancer-on-a-chip platform, which is a biomimetic approach to mimic the physiological tumor’s environment through seeding human cancer cells in a microfabricated platform in order to model the parameters, such as fluid shear force [19], concentration gradient [20], and a particular feature of TME including tumor and stromal cell interaction [21,22]. Given the merits of microfluidic cell culture platforms, these models enable the study of therapeutic agents that target key features of TME [21]. Herein, using a microfluidic model of tumor invasion using CAF-tumor spheroids, we demonstrated that targeting CAFs with PFD not only reduces EMT and invasion capacity of cancer cells but also decrease stemness by blocking the secretion of cytokines and the expression of YAP1 in breast carcinoma cells.

## 2. Materials and Methods

### 2.1. In-Silico Data Analysis

The TCGA breast cancer genomic information and clinical data were downloaded from the cBioportal data portal (https://www.cbioportal.org/ accessed on 25 March 2021) [23] and analyzed under Bioconductor tools in R-Software (version 3.8). A detailed description of used packages and related scripts to gene expression and mutation analysis is available at https://bioconductor.org/packages/release/bioc/vignettes/maftools/inst/doc/maftools.html accessed on 25 March 2021. The stromal index was calculated based on the ESTIMATE scoring method [24]. The EMT score in this study was calculated according to our previous study [25]. Protein–protein interaction analysis was performed with STRING [26,27].

### 2.2. Cell Lines and Media

Human breast adenocarcinoma cells, MCF7 and MDA-MB-231, were cultured in Dulbecco’s modified Eagle’s medium (DMEM) (Thermo Fisher Scientific, Waltham, MA, USA), supplemented with 10% (*v*/*v*) FBS, 100 U of penicillin/mL, and 100 µg of streptomycin/mL. The breast cancer-derived CAFs were cultured in DMEM supplemented with 1% (*v*/*v*) insulin–transferrin–selenium (ITS) (Thermo Fisher Scientific), 2% FBS (Thermo Fisher Scientific), and 1% penicillin/streptomycin (Sigma-Aldrich, St. Louis, MO, USA) in 37 °C.

### 2.3. Spheroid Formation

In order to obtain human tumor spheroids with stemness feature, the MCF7 cells were detached and suspended as individual cells at 1 × 10^5^ cells/mL in a Mammo Cult Human Medium Kit (STEMCELL Technology, Cat# 05620) included with hydrocortisone (STEMCELL Technology, Cat# 07925) and heparin solution (STEMCELL Technology, Cat# 07980), and cultured for 14 days onto a 100 mm ultra-low attachment dish (corning). To use the appropriate size of spheroids in the microfluidic device, the generated spheroids were collected and filtered in two consecutive filtration steps: (a) 40-μm filtration, in order to exclude all the spheroids smaller than 40 μm; and (b) 100-μm filtration to exclude aggregates larger than 100 μm and centrifuged by 250× *g* for 5 min to separate them from the supernatant. Dried plat containing spheroids were suspended in collagen Type-I solution on ice for loading in microfluidic devices [28].

### 2.4. Preparation of CAFs Condition Medium

CAFs were cultured at a concentration of 1 × 10^4^ cells/cm^2^ in DMEM medium supplemented with 1% (*v*/*v*) ITS and 1% (*v*/*v*) penicillin/streptomycin. Once the cells reached 95% confluence, cells were washed with PBS, and the culture media were replaced with serum and ITS-free DMEM followed by incubation for an additional 48 h. The concentrated supernatant was collected by centrifuging (Eppendorf, Hamburg, Germany)) at 1200 RPM for 5 min at room temperature, filtered through 0.45 µm filters, and designated as CAFs-conditioned medium (CAF-CM). The conditioned medium was then stored at −80 °C until use.

### 2.5. Microfluidic Device Design and Cell Culture

The microfluidic devices were purchased from AIM Biotech Company (Singapore). Each single device was composed of 2 side channels for loading cell culture medium and a central region channel for loading hydrogel containing cells. Next, 200 μL of collagen gel solution (2.5 mg/mL) at pH 7.4 was prepared on ice by the mixture of 20 μL of 10× PBS, 4 μL of NaOH (0.5 N), 129.2 μL of collagen Type-I (Corning, Cat# 354236.), 10 μL of cell suspension medium containing 1000–2000 tumor spheroids, and 22.9 μL of cell culture grade deionized water. After loading gel–cell solution into the central channel, the device was kept in the cell culture incubator at 37 °C and 5% CO2 for 40 min to allow gel polymerization. After polymerization, media channels filled with 120 μL of stemness medium or CAF-conditioned medium (CM), with or without PFD, by adding 70 µL in one port and another 50 µL into the opposite connected port of a media channel. This approach prevents shear stress on the gel channel following the loading medium.

For migration and co-culture study, the central channel was filled with a collagen gel solution (2.5 mg/mL) without embedded cells containing 20 μL of 10×PBS, 4μL of NaOH (0.5 N), 129.2 μL of collagen Type-I, and 42.8 μL of deionized water. Immediately after gel polymerization, the two side channels were filled with MDA-MB-231 (one side channel) and CAFs (opposite side channel) at the concentration of 10,000 cells for each cell line. Finally, 120 μL of the serum-free cell culture medium with or without PFD was added to the both side channels to feed the cells. In both experiments, the devices were kept in the incubator with the standard condition for four days.

### 2.6. Cytokine Profiling Assays on Treated samples

The culture medium of devices treated with PFD was analyzed using the Human Cytokine Antibody Array (Abcam, Cat# ab133997, Cambridge, UK) according to the provided protocol by manufacture. Briefly, the culture medium was collected from microfluidic devices and centrifuged followed by hybridization to the array membrane overnight at 4 °C. After washing the membrane, anti-cytokine secondary antibody was used. Finally, cytokines were detected by adding HRP-conjugated streptavidin on the membrane. The captured signals from each cytokine spots were quantified using ImageJ software.

### 2.7. Immunofluorescence Staining and Imaging Analysis

Cell culture media were removed from the devices, and samples in the microfluidic devices were first rinsed in 1X PBS by adding 70 µL of PBS into one port and another 50 µL into the opposite connected port of a media channel. Then, the cells were fixed with 4% paraformaldehyde (PFA) (Sigma-Aldrich, St. Louis, MO, USA) for 15 min at room temperature. Next, 0.1% Triton-X 100 (Sigma-Aldrich, St. Louis, MO, USA) was added, and the device was incubated for 10 min before blocking by BSA 1% (Sigma-Aldrich cat no.: A5611) for two hours, followed by staining of cells for α-SMA (1:100, Abcam, Cat# ab197240.), Vimentin (1:200, Biolegend Cat# 677804. San Diego, CA, USA), YAP1 (1:200, Abcam, Cat# ab205270), CD44 (1:200, Abcam, Cat# ab194988), and CDH1 (1:200, Biolegend Cat# 324104). Nikon Ti2 confocal microscopes were used for imaging of the samples. The intensity of fluorescent signal, an indicator of protein expression, was analyzed on multiple z-stack. Finally, the images’ mean fluorescent intensity (MFI) was quantified using Cell-Sense software (Olympus, Japan).

### 2.8. Statistical Analysis

The results of quantitative experiments were analyzed as mean ± SEM. The statistical analysis was performed with the Student *t*-test. * *p*-value < 0.05 was considered as a statistically significant and **** *p*-value < 0.0001 was considered as extremely significant. Microscopic images are representative images from three independent experiments.

## 3. Results

### 3.1. The Association of Tumor Stromal Content with the Expression of YAP1 and Metastasis Feature

**Figure 1:**
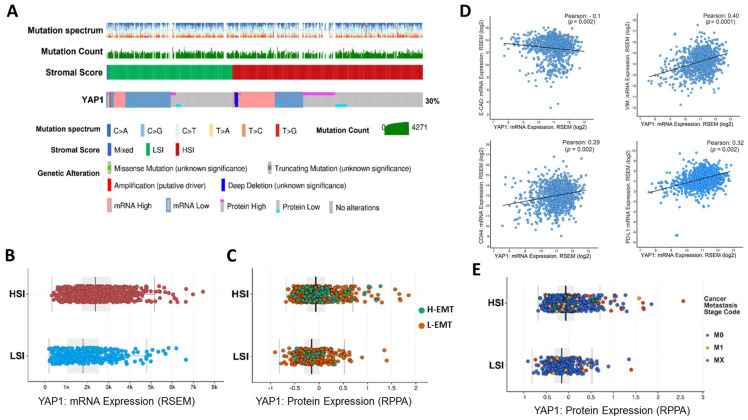
Genomic analysis of the TCGA breast cancer cohort. (**A**) The oncoprint illustrates YAP1 expression in two groups of samples annotated based on the stromal score signature. (**B**) Comparing the expression of YAP1 between samples with high-stromal index (HSI) and low-stromal index (LSI) indicating that samples with HSI expressed a high level of YAP1. (**C**) A High EMT status is enriched in samples with the HSI status and high level of YAP1 at the protein level. (**D**) Correlation coefficient analysis between the expression of YAP1 and classical EMT markers (VIM, CDH1), stemness marker (CD440, and immunosuppressive marker (PD-L1). The results show a negative association between YAP1 and CDH1 and a positive correlation with CD44, VIM, and PD-L1. (**E**) Association between the protein expression of YAP1 and metastatic stages in samples with a HSI and LSI score, showing enrichment of an adverse stage of metastasis in samples with high YAP1 expression and HSI score.

To better understand the potential association of YAP1 with classical stemness and EMT markers, we comprehensively analyzed the gene and protein expression levels of YAP1 across 1084 breast cancers from the TCGA cohorts. To evaluate the association between the expression of YAP1 and stromal content of TME, especially CAFs, we divided samples into two groups of the high-stromal index (HSI) and low-stromal index (LSI) according to the ESTIMATE score of samples (Figure 1). We observed a significantly high expression of YAP1 in samples with HSI scores compared to the LSI group (Figure 1A–C) at both transcriptome and protein levels. To study the association between YAP1 with EMT related features, we annotated HSI and LSI samples based on their EMT scores, namely a high EMT score (H-EMT) and a low EMT score (L-EMT), and measured expression of YAP1 across these groups at protein level (Figure 1C,D). Interestingly, we found that most of the samples with the HSI score include a H-EMT score sample compared to the LSI group.

Moreover, HSI samples represented high expression of YAP1 at the protein level (Figure 1C). To better understand whether there is an association between YAP1 and EMT, we analyzed the correlation coefficient between YAP1 and classical EMT genes, VIM and CDH1, plus stemness marker CD44 and immune checkpoint protein PD-L1. We found a positive association between YAP1 and VIM, CD44, and PD-L1, while YAP1 negatively correlated with CDH1 (Figure 1D).

Following these findings, we analyzed metastatic stages of individual samples across HSI and LSI groups and the prevalence of YAP1 expression. As depicted in Figure 1E, in comparison with the LSI group, most of the samples with the high expression of YAP1 at the protein level and a MX or M1 metastatic stage were enriched in the HSI group, indicating a positive correlation between stromal content, expression of YAP1, and high metastatic stage (Figure 1E). Taken together, these data highlight the positive association of high tumor stromal content of TME with the expression of YAP1 and induction of EMT and metastasis.

### 3.2. Association of YAP1 with EMT and Stemness Markers

**Figure 2:**
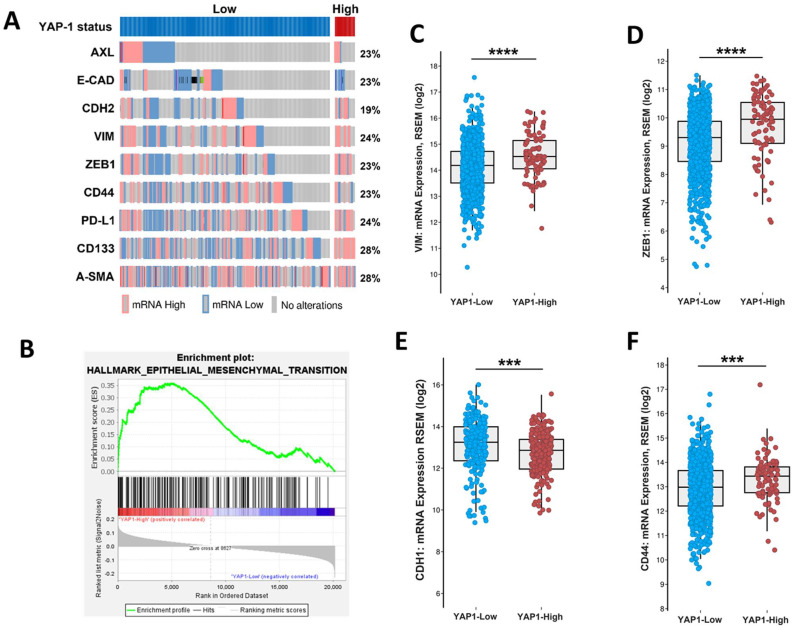
In silico data analysis between samples expressing high and low levels of YAP1. (**A**) The oncoprint plot depicts the high expression of EMT- and stemness-related genes in samples with YAP1-high status at the transcriptome level. (**B**) The gene-set enrichment analysis (GSEA) shows the enrichment of hallmarks of EMT in samples with a high level of YAP1. (**C**–**F**) The comparison between high and low YAP1 groups in terms of VIM expression (**C**), ZEB1 (**D**), CDH1 (**E**), and CD44 (**F**). *** *p* < 0.0002, **** *p* < 0.0001 using student *t*-test.

To further demonstrate of association between YAP1 and EMT, we classified samples into two groups of YAP1-high and YAP1-low, based on its gene expression level. The oncoprint analysis illustrated enrichment high expression of EMT and stemness-related genes in samples with high expression of YAP1 (Figure 2A). Moreover, the gene-set enrichment analysis (GSEA) of YAP1-low and -high samples resulted in the enrichment of hallmark of EMT in YAP1-high samples in comparison with YAP1-low (Figure 2B), in which the expression of classical EMT markers VIM and ZEB1 were significantly higher across YAP1-high samples (Figure 2C,D). In contrast, CDH1 expression was enriched in the YAP1-low group (Figure 2E). Additionally, samples with the high expression of YAP1 represented a significant expression of stemness marker CD44 compared to the YAP1-low expressing samples (Figure 2F).

### 3.3. CAFs Induce Spheroid Formation

**Figure 3:**
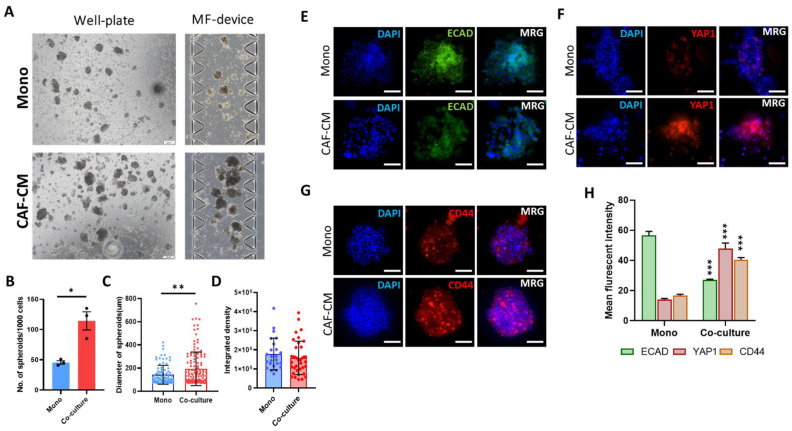
CAFs induces spheroid formation with increased diameter and expression of YAP1. (**A**) Bright-filed snapshot image of the formation of spheroids in well-plate (left) and microfluidic device (right) co-cultured with or without CAF-CM. The micrographs show an increase in the number and size of generated spheroids on both platforms in the presence of CAF-CM. (Scale bar: 200 µm) (**B**) The CAF-CM significantly increased the formation of spheroids per 1000 cells in comparison with monoculture. (**C**) In comparison with monoculture, co-culturing cancer cells with CAF-CM significantly increased the size of generated spheroids. (**D**) The integrated density in spheroids co-cultured with CAF-CM was lower than in monoculture, indicating the stimulation of cell invasion from spheroids in the ECM channel. (**E**–**G**) The immunofluorescence staining of the EMT marker ECAD (**E**) and stemness marker CD44 (**F**) indicates that CAF-CM stimulates EMT and stemness. Co-culturing with CAF-CM increased the expression of YAP1 in generated spheroids (Scale bar: 100 µm). (**H**) The quantitative analysis of mean fluorescence intensity in ECAD, YAP1, and CD44 resulted in a significant reduction in the expression of ECAD and an increase in CD44 and YAP1 in the co-culture group to the monoculture. * *p* < 0.05, ** *p* < 0.002, *** *p* < 0.0002 using the Student *t*-test.

To investigate whether CAFs can induce and stimulate spheroid formation, we generated a concentrated condition medium from CAFs (CAF-CM) and co-cultured MCF7 cells in well-plate and microfluidic devices in 50:50 (*v*/*v*) ratio of CAF-CM and stemness induction medium for seven days. We found that the number and the diameter of generated spheroids were significantly increased when the cells were co-cultured with CAF-CM (Figure 3A–C), while integrated density properties of spheroids were reduced after seven days in comparison with the monoculture group (Figure 3D). The assessment of the epithelial marker E-cadherin showed that CAF-CM significantly reduced expression of this gene, suggesting that CAF-CM may induce migration of cells in spheroids (Figure 3E,H). Moreover, we found a significant expression on YAP1 (Figure 3F,H) and CD44 in spheroids co-cultured with CAF-CM compared to the monoculture samples (Figure 3G,H). These data indicate an invasion and stemness induction role of CAF cells through the secretion of cytokines.

### 3.4. Pirfenidone Reduces the Migration of CAFs and Carcinoma Cells

**Figure 4:**
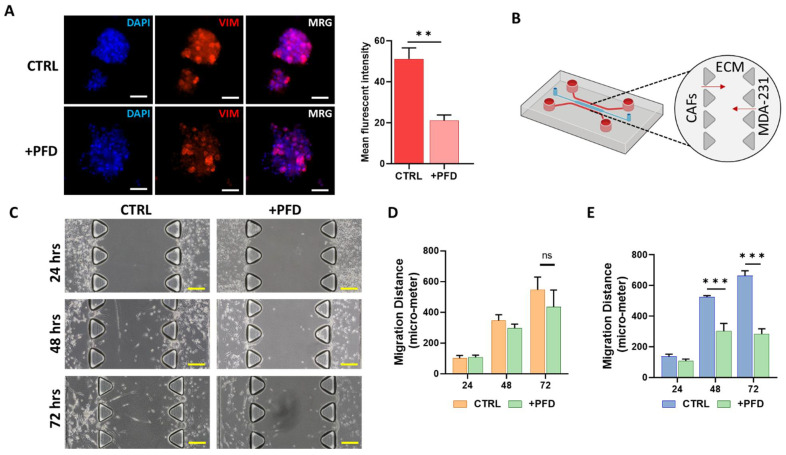
Assessing the migration and invasion inhibitory effects of PFD on MDA-MB-231 carcinoma cells and CAFs. (**A**) Tumor spheroids derived from MDA-MB-231 show a significant reduction in the expression of EMT marker VIM (scale bar: 100 µm). (**B**) The schematic image of invasion and migration assay performed in the 3D microfluidic device in which MDA-MB-231 and CAFs are injected in side channels. The migration of cells was monitored for three days after seeding. (**C**) A representative bright-field image of migration of CAFs and MDA-MB-231 in the presence and absence of PFD highlight that PFD significantly suppressed the migration of both cells during 72 h. (scale bar: 200 µm). (**D**,**E**) The quantitative analysis of migration distance for CAFs (**D**) and MDA-MB-231 cells (**E**) indicated that PFD did not significantly suppress the migration of CAFs after 72 h; however, in comparison with the control group, a reduction was observed. In the case of MDA-MB-231 cells, PFD significantly suppressed the invasion and migration of these cells after 48 and 72 h. ** *p* < 0.002 using the Student *t*-test, *** *p* < 0.0002 using ANOVA.

The role of tumor stromal cells, particularly CAFs, in the induction of migration in carcinoma cells has been shown previously [29]. To study the potential of PFD in reducing invasive properties of carcinoma cells, we first co-cultured MCF7 carcinoma cells known as low-vimentin expressers with CAF-CM for 48 h and then treated cells with a low concentration of PFD (40 µM) for 72 h (Figure 4A). In comparison with the control group, we observed a significant reduction in vimentin expression in spheroids treated with PFD (Figure 4A). Following these results, we assessed the effects of PFD on the migration of carcinoma cells and CAFs. To this aim, we co-cultured CAFs and MDA-MB-231 as representative of aggressive breast cancer in the 3D microfluidic device and treated cells with 40 µM of PFD for 72 h, followed by measuring cell migration distance in control and PFD-treated cells (Figure 4B). Figure 4C presents a snapshot of the 3D device including migration of CAFs and MDA-MB-231 through the ECM channel in three different time points. In PFD-treated samples, we loaded serum-free culture medium containing (40 µM) PFD following seeding cells in the device.

Interestingly, we found that the presence of PFD in the culture medium significantly reduced the migration of MDA-MB-231 cells (Figure 4E) after 48 and 72 h. In the case of CAFs, although migration inhibitory of PFD did not reach statistical significance, we observed a reduction in migration of CAFs through the ECM after 72 h compared to the control group. These data highlight the potential of PFD to reduce the invasion and migration capacity of carcinoma cells and CAFs.

### 3.5. Pirfenidone Reduces YAP1 and CD44 Expression in Spheroids by Targeting Key Cytokines

**Figure 5:**
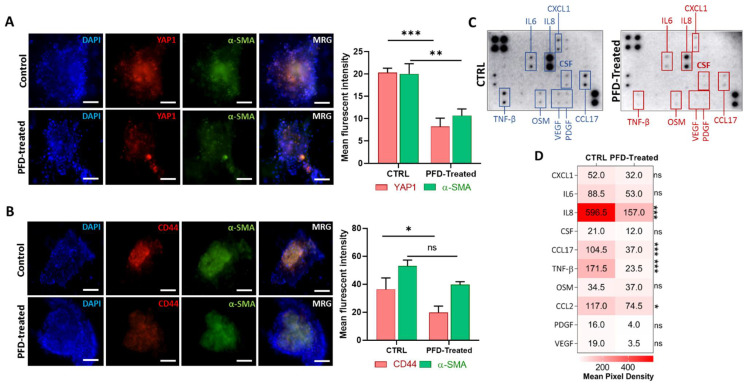
PFD reduces CAFs activity and expression of CD44 and YAP1 through the blockade of key cytokine secretion. (**A**) The immunofluorescence staining shows a significant decrease in protein expression of YAP1 and α-SMA following treatment with PFD (scale bar: 100 µm). (**B**) Besides α-SMA, PFD also reduced the expression of CD44 in spheroids that include CAFs (scale bar: 100 µm). (**C**) The picture of cytokine profiling performed on culture medium retrieved from PFD-treated and non-treated spheroids in devices. The array shows the depletion of numerous cytokines in treated samples. (**D**) The quantitative analysis of the mean-pixel density analysis of cytokine membranes using ImageJ software, shows a significant depletion of IL6, IL8, CCL17, TNF-β. * *p* < 0.05, ** *p* < 0.002, *** *p* < 0.0002, using Student *t*-test (**A**,**B**) and ANOVA (**D**).

The cytokine production inhibitory role of PFD has been shown in various studies [15,28]. Targeting cancer progression-promoting cytokines using PFD may reduce stemness and immune suppression promoted by CAFs. To study the effect of PFD on the expression of stemness marker CD44 and also of YAP1, we first cultured MCF7 cells with CAF-CM for 72 h and then generated spheroids that include both MCF7 and CAFs in a ratio of 2:1 and cultured them into the 3D device. The generated spheroids were treated with stemness medium containing 40 µM of PFD for 72 h before performing imaging and cytokine profiling. Interestingly, we found that PFD not only significantly reduced the expression of YAP1 (Figure 5A) and CD44 (Figure 5C) but also the level of α-SMA after treatment, indicating a reduction in CAFs activity in spheroids (Figure 5A,B). To better understand the inhibitory effects of PFD, we collected culture medium from 3D microfluidic devices from both groups after 72 h and performed a cytokine profiling array against 42 cancer-promoting cytokines (Figure 5C). A significant reduction was observed in the secretion of various cytokines after treatment with PFD, including IL8, CCL17, TNF-β, and CCL2 (Figure 5D).

**Figure 6:**
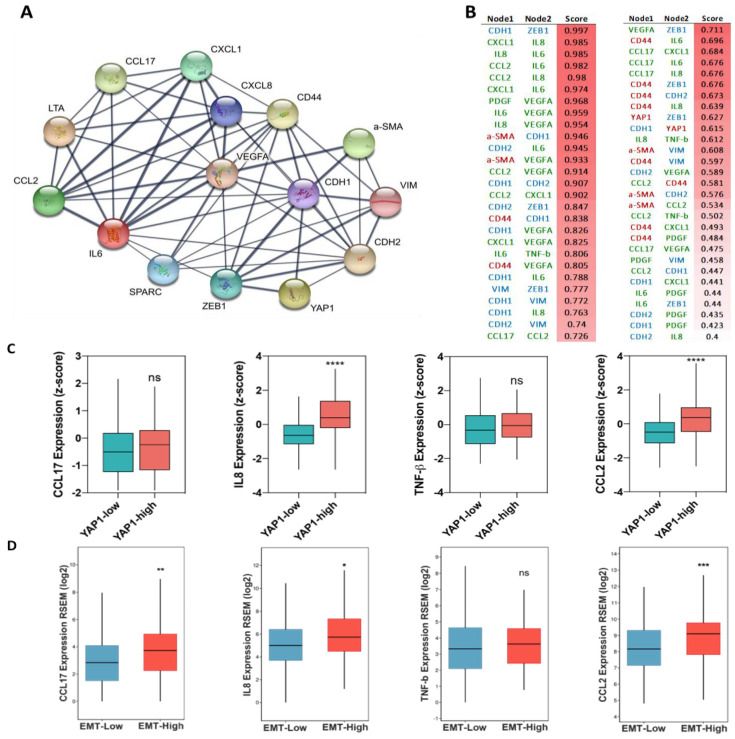
The association between targeted cytokines with EMT and YAP1. (**A**) The graphical image of the network between related genes, showing an association between EMT, stemness, and YAP1. (**B**) Scoring results between nodes in the PPI network. (**C**) Comparison between the expression of four identified cytokines in samples with high- and low expression of YAP1 in breast cancer indicating enrichment of these cytokines in YAP1-high samples. (**D**) Key cytokines are enriched in samples with high EMT score. * *p* < 0.05, ** *p* < 0.002, *** *p* < 0.0002, **** *p* < 0.0001, using the Student *t*-test.

Moreover, we observed a slight decrease in the level of CXCL1, IL6, CSF, OSM, PDGF, and VEGF in treated samples as compared to the control group (Figure 5D). The protein–protein interaction analysis between classical EMT genes, stemness, and PFD targeted cytokines revealed associations between YAP1 and CD44 with VIM, CDH1, and AXL and IL8 (Figure 6A,B). In order to further evaluate, the association between identified cytokines with EMT and YAP1, we analyzed expression of these cytokines in samples with high and low expression of YAP1 from TCGA breast cancer cohort. As illustrated in Figure 6C, a significant expression of IL8 and CCL2 was enriched in samples with high YAP1 level. Besides, although the YAP1-high samples showed the high expression of CCL17 and TNF-β in comparison with YAP1-low, however, significant difference was not observed between these groups. (Figure 6C). Following these findings, we analyzed EMT scores across 1084 samples from TCGA and classified samples into high and low EMT scores and assessed the expression of identified cytokines. Interestingly, we found the significant enrichment of targeted cytokines in samples with a high EMT score compared to the low EMT score samples, indicating a positive association between expression of targeted cytokines and EMT in breast cancer (Figure 6D). These results indicate that PFD might reduce the stemness and invasion capacity of the carcinoma cells through targeting cytokine production in CAF that regulate EMT, which in turn can stimulate YAP1-associated stemness.

## 4. Discussion

The role of cellular components of TME, especially CAFs, in promoting carcinoma progression is well understood [3,8]. Numerous studies highlighted that CAFs could induce stemness features in carcinoma cells through the secretion of various cytokines and remodeling ECM toward developing a high stiffness of ECM which, in turn, stimulates phenotypic changes including EMT and induces the expression of related stemness genes [8,30,31,32,33]. Targeting CAFs to either suppress their activity or reprogram them into an inactive state showed some promising effects [34]. Inhibiting carcinoma progression-promoting cytokines released from CAFs may be an efficacious therapeutic strategy for cancer treatment [35,36,37].

Recently, various clinical and preclinical studies demonstrated the potential application of PFD as a combination therapy with chemotherapy and radiotherapy in various types of cancers, including lung and pancreatic carcinoma [13,37]. However, few studies showed PFD application in suppressing invasive properties, particularly EMT, and reduction in carcinoma-stemness through the targeting of key cytokines and genes that regulate ECM synthesis and remodeling [38,39,40]. As a proof-of-concept study, we hypothesized that PFD could reduce CAF-induced EMT and consequently EMT-regulated stemness in breast carcinoma cells by depletion of key cytokines.

Using a comprehensive in-silico data analysis on the breast cancer cohort from TCGA, we showed the enrichment of high-level YAP1 expression in samples with the highest stromal score. Moreover, we illustrated that samples with high stromal scores exhibit a high score of EMT and invasive properties. A positive association between high level of YAP1 and EMT-related genes, including vimentin and ZEB1 and stemness genes, at both transcriptomic and protein levels was observed [41,42]. In line with these findings, several studies demonstrated that YAP1 is a mediator of EMT and stemness in numerous types of carcinoma, including breast cancer [42,43,44,45].

Tumor stromal cells, particularly CAFs, are among the main modulator of EMT and stemness [46] through the induction of YAP1 and secretion of various cytokines. The strategy based on targeting these proteins directly or indirectly by targeting CAFs might be a promising approach to the treatment of advanced carcinoma [36,39]. To model and study the cellular interaction of TME, particularly CAFs, and the effects of stromal cells in the migration of cancer cells, various in-vitro models have been developed ranging from co-culture 2D platforms to 3D cell-culture models [47,48], including tumor organoids. However, the drawbacks of each model limited their application as a reliable and low-cost model for both TME analysis and drug screening [49]. To overcome these limitations, microfluidic 3D cell-culture platforms offered a reliable and physiologically relevant model to study and mimic particular features of TME, especially tumor–stromal cell interaction in a fully controlled setting. Using low input material, controlling over culture compared to current 3D models, high capability of real-time imaging, and the possibility of co-culturing tumor organoids with other components of TME, including stromal cells and immune cells, enable this platform to model cellular interaction of TME in a reliable fashion [21]. Moreover, the design of microfluidic devices allows the modelling of cellular infiltration and compound diffusion in the TME site [50,51].

In this study, using a 3D microfluidic platform, we modelled the reciprocal interaction between CAFs and breast carcinoma cells. Using our invasion and migration model, we demonstrated that CAFs promote spheroid formation and the migration capacity of carcinoma cells [15], possibly through the secretion of cancer-promoting cytokines that play a haptotactic or chemotactic role for carcinoma cells [52]. Following treatment with PFD, we found that PFD significantly reduced the migration of an invasive type of breast cancer cells and CAFs, and significantly decreased the expression of EMT marker vimentin, stemness marker CD44, and YAP1 in CAF-tumor spheroids at the protein level [41]. Similar observations were reported previously for the migratory inhibition effect of PFD on both carcinoma cells and CAFs in pancreatic cancer [13] and breast cancer [15,53]. The role of matrix-metalloproteinases (MMPs) on the stimulation of invasion and angiogenesis is well established [54]. It has been shown that MMP-9 and MMP-2 released from CAFs can stimulate the potential of invasion and migration in carcinoma cells [55,56]. Through our invasion model, we demonstrated the migration inhibitory effect of CAFs. These data suggest that, besides EMT genes, PFD may suppress invasion by depletion of particular MMP in both CAFs and cancer cells.

As the expression of stemness marker CD44 on cancer cells is linked to the hypoxic condition, recent studies highlighted that hypoxic and hypo-nutritional conditions might induce the expression of CD44 on CAFs as well [57,58]; however, the validation of these findings require further studies in the broad range of cancer types. Although we used a non-hypoxic condition in our model, the effect of PFD on the reduction in CD44 on breast carcinoma cells suggests that further investigation is required on the stemness inhibitory role of PFD on a hypoxic model of TME.

In this study, we measured the expression of vimentin, ZEB1, and E-cadherin as major EMT-related markers in our generated spheroids in microfluidic device. The protein–protein interaction analysis also resulted in an association between other EMT-related genes, including N-cadherin with YAP1, CD44, and α-SMA, suggesting that these genes may play a dual role in both the activation of CAFs and stemness in carcinoma cells [59].

Recent single-cell analysis studies revealed that a sub-population of CAFs, called inflammatory CAFs, express IL6 and TNF-α/β [60]. The co-culture of colon tumor organoids with this population of CAFs resulted in the upregulation of vimentin and ZEB1 in tumor cells [60]. Moreover, CAF-derived cytokines significantly induce the expression of HOTAIR, which in return promotes the EMT program in metastatic breast cancers [53]. This study also showed that targeting CAFs with PFD blocks the TGF-β1/HOTAIR axis and decreases the migration potential of MDA-MB-231 cells. In addition to these findings, we found that the level of α-SMA decreased after treatment with PFD, indicating that PFD can reduce CAFs activity [34]. In line with these observations, numerous in-vitro studies highlighted the inactivity of CAFs after treatment with PFD [15,28].

Using cytokine profiling, we demonstrated that PFD significantly depleted the secretion of cancer- and invasion-promoting cytokines, particularly IL8, CCL17, and TNF-β. Many studies highlighted the critical role of these factors in the interaction of the carcinoma–stromal cell and ECM deposition toward the induction of an invasive TME and activation of EMT and stemness [6,7,61,62].

It has been shown that the secretion of IL8 by CAFs, known as EMT and stemness modulator [63], and tumor-associated mesenchymal stem cells (TA-MSCs) not only induced the migration of carcinoma cells but also up-regulated immune suppression in carcinoma cells through the PD-L1 expression. The treatment of TAMs with PFD reduced expression of PD-L1 and migration of carcinoma cells in in vitro models [28]. CCL17 was reported as a key cytokine in the generation of tumor-associated macrophages [64], which stimulates EMT and stemness [65,66]. Moreover, it has been shown that the production and secretion of CCL17 within the TME through the CAFs trigger the recruitment of myeloid-derived suppressor cells (MDSCs) to the TME, promoting invasion in various cancers [67,68,69]. For instance, recently, Omland and colleagues reported that CCL17 secreted from resident CAFs within cutaneous basal cell carcinoma TME increases tumor progression and amplifies immune suppression [70].

Taken together, in line with numerous studies, the results reported in this study highlight the importance of suppressing CAF-derived cytokines that can induce both EMT and stemness. This study also emphasizes the need to further explore the cross-talks between carcinoma cells and other cells of the TME to better control tumor progression.

## 5. Conclusions

Targeting CAFs has a great potential in treating highly aggressive types of cancers, including breast carcinoma. The PFD, as a well-known anti-fibrotic agent, can suppress the activation of fibroblasts through targeting cytokines. In this study, using a microfluidic culture platform, we demonstrated the inhibitory function of PFD on invasion, EMT, and stemness characteristics of invasive breast cancer cells through the depletion of cytokines. However, further in vivo investigation is needed to delineate the potential of PFD as a treatment modality in combination with current therapeutic agents in the treatment of metastatic cancers.

## Data Availability

The genomic data from the TCGA-Pan Breast Carcinoma dataset used in this study are openly available for download in (www.cBioportal.org, Accessed date: 25 March 2021). All data generated and described in this article are available from the corresponding web servers and portal and are freely available to download for noncommercial purposes, without breaching participant confidentiality.

## References

[1] Prager B.C., Xie Q., Bao S., Rich J.N. (2019). Cancer Stem Cells: The Architects of the Tumor Ecosystem. Cell Stem Cell.

[2] Ferguson L.P., Diaz E., Reya T. (2021). The Role of the Microenvironment and Immune System in Regulating Stem Cell Fate in Cancer. Trends Cancer.

[3] Kalluri R. (2016). The biology and function of fibroblasts in cancer. Nat. Rev. Cancer.

[4] Wu S.Z., Roden D.L., Wang C., Holliday H., Harvey K., Cazet A.S., Murphy K.J., Pereira B., Al-Eryani G., Bartonicek N. (2020). Stromal cell diversity associated with immune evasion in human triple-negative breast cancer. EMBO J..

[5] Fiori M.E., Di Franco S., Villanova L., Bianca P., Stassi G., De Maria R. (2019). Cancer-associated fibroblasts as abettors of tumor progression at the crossroads of EMT and therapy resistance. Mol. Cancer.

[6] Yu Y., Xiao C.H., Tan L., Wang Q.S., Li X.Q., Feng Y. (2014). Cancer-associated fibroblasts induce epithelial-mesenchymal transition of breast cancer cells through paracrine TGF-beta signalling. Br. J. Cancer.

[7] Shintani Y., Fujiwara A., Kimura T., Kawamura T., Funaki S., Minami M., Okumura M. (2016). IL-6 Secreted from Cancer-Associated Fibroblasts Mediates Chemoresistance in NSCLC by Increasing Epithelial-Mesenchymal Transition Signaling. J. Thorac. Oncol..

[8] Cox T.R. (2021). The matrix in cancer. Nat. Rev. Cancer.

[9] Shibue T., Weinberg R.A. (2017). EMT, CSCs, and drug resistance: The mechanistic link and clinical implications. Nat. Rev. Clin. Oncol..

[10] Zanconato F., Cordenonsi M., Piccolo S. (2019). YAP and TAZ: A signalling hub of the tumour microenvironment. Nat. Rev. Cancer.

[11] Sahai E., Astsaturov I., Cukierman E., DeNardo D.G., Egeblad M., Evans R., Fearon D., Greten F.R., Hingorani S.R., Hunter T. (2020). A framework for advancing our understanding of cancer-associated fibroblasts. Nat. Rev. Cancer.

[12] Polydorou C., Mpekris F., Papageorgis P., Voutouri C., Stylianopoulos T. (2017). Pirfenidone normalizes the tumor microenvironment to improve chemotherapy. Oncotarget.

[13] Kozono S., Ohuchida K., Eguchi D., Ikenaga N., Fujiwara K., Cui L., Mizumoto K., Tanaka M. (2013). Pirfenidone Inhibits Pancreatic Cancer Desmoplasia by Regulating Stellate Cells. Cancer Res..

[14] Li C., Rezov V., Joensuu E., Vartiainen V., Rönty M., Yin M., Myllã¤Rniemi L.M., Koli K. (2018). Pirfenidone decreases mesothelioma cell proliferation and migration via inhibition of ERK and AKT and regulates mesothelioma tumor microenvironment in vivo. Sci. Rep..

[15] Aboulkheyr Es H., Zhand S., Thiery J.P., Warkiani M.E. (2020). Pirfenidone reduces immune-suppressive capacity of cancer-associated fibroblasts through targeting CCL17 and TNF-beta. Integr. Biol..

[16] Nii T., Makino K., Tabata Y. (2019). A Cancer Invasion Model Combined with Cancer-Associated Fibroblasts Aggregates Incorporating Gelatin Hydrogel Microspheres Containing a p53 Inhibitor. Tissue Eng. Part C Methods.

[17] Nguyen M., De Ninno A., Mencattini A., Mermet-Meillon F., Fornabaio G., Evans S.S., Cossutta M., Khira Y., Han W., Sirven P. (2018). Dissecting Effects of Anti-cancer Drugs and Cancer-Associated Fibroblasts by On-Chip Reconstitution of Immunocompetent Tumor Microenvironments. Cell Rep..

[18] Nii T., Makino K., Tabata Y. (2020). Three-Dimensional Culture System of Cancer Cells Combined with Biomaterials for Drug Screening. Cancers.

[19] Galie P.A., Nguyen D.-H.T., Choi C.K., Cohen D.M., Janmey P.A., Chen C.S. (2014). Fluid shear stress threshold regulates angiogenic sprouting. Proc. Natl. Acad. Sci. USA.

[20] Byrne M.B., Leslie M.T., Gaskins H.R., Kenis P.J. (2014). Methods to study the tumor microenvironment under controlled oxygen conditions. Trends Biotechnol..

[21] Sontheimer-Phelps A., Hassell B.A., Ingber D.E. (2019). Modelling cancer in microfluidic human organs-on-chips. Nat. Rev. Cancer.

[22] Skardal A., Murphy S.V., Devarasetty M., Mead I., Kang H.-W., Seol Y.-J., Zhang Y.S., Shin S.-R., Zhao L., Aleman J. (2017). Multi-tissue interactions in an integrated three-tissue organ-on-a-chip platform. Sci. Rep..

[23] Gao J., Aksoy B.A., Dogrusoz U., Dresdner G., Gross B., Sumer S.O., Sun Y., Jacobsen A., Sinha R., Larsson E. (2013). Integrative Analysis of Complex Cancer Genomics and Clinical Profiles Using the cBioPortal. Sci. Signal..

[24] Yoshihara K., Shahmoradgoli M., Martínez E., Vegesna R., Kim H., Torres-Garcia W., Trevino V., Shen H., Laird P.W., Levine D.A. (2013). Inferring tumour purity and stromal and immune cell admixture from expression data. Nat. Commun..

[25] Tan T.Z., Miow Q.H., Miki Y., Noda T., Mori S., Huang R.Y.-J., Thiery J.P. (2014). Epithelial-mesenchymal transition spectrum quantification and its efficacy in deciphering survival and drug responses of cancer patients. EMBO Mol. Med..

[26] Snel B., Lehmann G., Bork P., Huynen M.A. (2000). STRING: A web-server to retrieve and display the repeatedly occurring neighbourhood of a gene. Nucleic Acids Res..

[27] Szklarczyk D., Gable A.L., Nastou K.C., Lyon D., Kirsch R., Pyysalo S., Doncheva N.T., Legeay M., Fang T., Bork P. (2021). Correction to ‘The STRING database in 2021: Customizable protein–protein networks, and functional characterization of user-uploaded gene/measurement sets’. Nucleic Acids Res..

[28] Es H.A., Bigdeli B., Zhand S., Aref A.R., Thiery J.P., Warkiani M.E. (2020). Mesenchymal stem cells induce PD-L1 expression through the secretion of CCL5 in breast cancer cells. J. Cell. Physiol..

[29] Wang Z., Liu J., Huang H., Ye M., Li X., Wu R., Liu H., Song Y. (2021). Metastasis-associated fibroblasts: An emerging target for metastatic cancer. Biomark. Res..

[30] Zanconato F., Cordenonsi M., Piccolo S. (2016). YAP/TAZ at the Roots of Cancer. Cancer Cell.

[31] Wilson M.M., Weinberg R.A., Lees J.A., Guen V.J. (2020). Emerging Mechanisms by which EMT Programs Control Stemness. Trends Cancer.

[32] Balachander G.M., Talukdar P.M., Debnath M., Rangarajan A., Chatterjee K. (2018). Inflammatory Role of Cancer-Associated Fibroblasts in Invasive Breast Tumors Revealed Using a Fibrous Polymer Scaffold. ACS Appl. Mater. Interfaces.

[33] Nallanthighal S., Heiserman J.P., Cheon D.-J. (2019). The Role of the Extracellular Matrix in Cancer Stemness. Front. Cell Dev. Biol..

[34] Costa A., Kieffer Y., Scholer-Dahirel A., Pelon F., Bourachot B., Cardon M., Sirven P., Magagna I., Fuhrmann L., Bernard C. (2018). Fibroblast Heterogeneity and Immunosuppressive Environment in Human Breast Cancer. Cancer Cell.

[35] Sharma M., Turaga R.C., Yuan Y., Satyanarayana G., Mishra F., Bian Z., Liu W., Sun L., Yang J., Liu Z.-R. (2021). Simultaneously targeting cancer-associated fibroblasts and angiogenic vessel as a treatment for TNBC. J. Exp. Med..

[36] Chen X., Song E. (2018). Turning foes to friends: Targeting cancer-associated fibroblasts. Nat. Rev. Drug Discov..

[37] Fujiwara A., Funaki S., Fukui E., Kimura K., Kanou T., Ose N., Minami M., Shintani Y. (2020). Effects of pirfenidone targeting the tumor microenvironment and tumor-stroma interaction as a novel treatment for non-small cell lung cancer. Sci. Rep..

[38] Kurimoto R., Ebata T., Iwasawa S., Ishiwata T., Tada Y., Tatsumi K., Takiguchi Y. (2017). Pirfenidone may revert the epithelial-to-mesenchymal transition in human lung adenocarcinoma. Oncol. Lett..

[39] Takai K., Le A., Weaver V.M., Werb Z. (2016). Targeting the cancer-associated fibroblasts as a treatment in triple-negative breast cancer. Oncotarget.

[40] Marwitz S., Turkowski K., Nitschkowski D., Weigert A., Brandenburg J., Reiling N., Thomas M., Reck M., Drömann D., Seeger W. (2020). The Multi-Modal Effect of the Anti-fibrotic Drug Pirfenidone on NSCLC. Front. Oncol..

[41] Liu Y., Siles L., Lu X., Dean K.C., Cuatrecasas M., Postigo A., Dean D.C. (2018). Mitotic polarization of transcription factors during asymmetric division establishes fate of forming cancer cells. Nat. Commun..

[42] Shao D., Xue W., Krall E.B., Bhutkar A., Piccioni F., Wang X., Schinzel A.C., Sood S., Rosenbluh J., Kim J.W. (2014). KRAS and YAP1 Converge to Regulate EMT and Tumor Survival. Cell.

[43] Yu M., Chen Y., Xuelian L., Yang R., Zhang L., Huangfu L., Zheng N., Zhao X., Lv L., Hong Y. (2018). YAP1 contributes to NSCLC invasion and migration by promoting Slug transcription via the transcription co-factor TEAD. Cell Death Dis..

[44] Wang T., Mao B., Cheng C., Zou Z., Gao J., Yang Y., Lei T., Qi X., Yuan Z., Xu W. (2018). YAP promotes breast cancer metastasis by repressing growth differentiation factor-15. Biochim. Biophys. Acta (BBA)-Mol. Basis Dis..

[45] Li S., Zhu H., Chen H., Xia J., Zhang F., Xu R., Lin Q. (2020). Glucose promotes epithelial-mesenchymal transitions in bladder cancer by regulating the functions of YAP1 and TAZ. J. Cell. Mol. Med..

[46] Giannoni E., Bianchini F., Calorini L., Chiarugi P. (2011). Cancer Associated Fibroblasts Exploit Reactive Oxygen Species Through a Proinflammatory Signature Leading to Epithelial Mesenchymal Transition and Stemness. Antioxid. Redox Signal..

[47] Campbell J.J., Husmann A., Hume R.D., Watson C.J., Cameron R.E. (2017). Development of three-dimensional collagen scaffolds with controlled architecture for cell migration studies using breast cancer cell lines. Biomaterials.

[48] Liu C., Liu Y., Xu X.-X., Guo X., Sun G.-W., Ma X.-J. (2016). Mesenchymal stem cells enhance the metastasis of 3D-cultured hepatocellular carcinoma cells. BMC Cancer.

[49] Garreta E., Kamm R.D., Lopes S.M.C.D.S., Lancaster M.A., Weiss R., Trepat X., Hyun I., Montserrat N. (2020). Rethinking organoid technology through bioengineering. Nat. Mater..

[50] Haase K., Offeddu G.S., Gillrie M.R., Kamm R.D. (2020). Endothelial Regulation of Drug Transport in a 3D Vascularized Tumor Model. Adv. Funct. Mater..

[51] Hajal C., Ibrahim L., Serrano J.C., Offeddu G.S., Kamm R.D. (2020). The effects of luminal and trans-endothelial fluid flows on the extravasation and tissue invasion of tumor cells in a 3D in vitro microvascular platform. Biomaterials.

[52] Mukaida N., Zhang D., Sasaki S.-I. (2020). Emergence of Cancer-Associated Fibroblasts as an Indispensable Cellular Player in Bone Metastasis Process. Cancers.

[53] Ren Y., Jia H.-H., Xu Y.-Q., Zhou X., Zhao X.-H., Wang Y., Song X., Zhu Z.-Y., Sun T., Dou Y. (2018). Paracrine and epigenetic control of CAF-induced metastasis: The role of HOTAIR stimulated by TGF-ß1 secretion. Mol. Cancer.

[54] Quintero-Fabián S., Arreola R., Villanueva L.E.B., Torres-Romero J., Arana-Argáez V., Lara-Riegos J., Ramírez-Camacho M.A., Alvarez-Sánchez M.E. (2019). Role of Matrix Metalloproteinases in Angiogenesis and Cancer. Front. Oncol..

[55] Sáenz-De-Santa-María I., Celada L., Martínez A.S.J., Cubiella T., Chiara M.-D. (2020). Blockage of Squamous Cancer Cell Collective Invasion by FAK Inhibition Is Released by CAFs and MMP-2. Cancers.

[56] Taguchi A., Kawana K., Tomio K., Yamashita A., Isobe Y., Nagasaka K., Koga K., Inoue T., Nishida H., Kojima S. (2014). Matrix Metalloproteinase (MMP)-9 in Cancer-Associated Fibroblasts (CAFs) Is Suppressed by Omega-3 Polyunsaturated Fatty Acids In Vitro and In Vivo. PLoS ONE.

[57] Kinugasa Y., Matsui T., Takakura N. (2013). CD44 Expressed on Cancer-Associated Fibroblasts Is a Functional Molecule Supporting the Stemness and Drug Resistance of Malignant Cancer Cells in the Tumor Microenvironment. STEM CELLS.

[58] Zhan Y., Du J., Min Z., Ma L., Zhang W., Zhu W., Liu Y. (2021). Carcinoma-associated fibroblasts derived exosomes modulate breast cancer cell stemness through exonic circHIF1A by miR-580-5p in hypoxic stress. Cell Death Discov..

[59] Pelon F., Bourachot B., Kieffer Y., Magagna I., Mermet-Meillon F., Bonnet I., Costa A., Givel A.-M., Attieh Y., Barbazan J. (2020). Cancer-associated fibroblast heterogeneity in axillary lymph nodes drives metastases in breast cancer through complementary mechanisms. Nat. Commun..

[60] Mosa M.H., Michels B.E., Menche C., Nicolas A.M., Darvishi T., Greten F.R., Farin H.F. (2020). A Wnt-Induced Phenotypic Switch in Cancer-Associated Fibroblasts Inhibits EMT in Colorectal Cancer. Cancer Res..

[61] Lamouille S., Xu J., Derynck R. (2014). Molecular mechanisms of epithelial–mesenchymal transition. Nat. Rev. Mol. Cell Biol..

[62] Yeow Y.L., Kotamraju V.R., Wang X., Chopra M., Azme N., Wu J., Schoep T.D., Delaney D.S., Feindel K., Li J. (2019). Immune-mediated ECM depletion improves tumour perfusion and payload delivery. EMBO Mol. Med..

[63] Su S., Chen J., Yao H., Liu J., Yu S., Lao L., Wang M., Luo M., Xing Y., Chen F. (2018). CD10(+)GPR77(+) Cancer-Associated Fibroblasts Promote Cancer Formation and Chemoresistance by Sustaining Cancer Stemness. Cell.

[64] Casagrande N., Borghese C.P., Visser L., Mongiat M., Colombatti A., Aldinucci D. (2018). CCR5 antagonism by maraviroc inhibits Hodgkin lymphoma microenvironment interactions and xenograft growth. Haematologica.

[65] Fan Q.M., Jing Y.Y., Yu G.F., Kou X.R., Ye F., Gao L., Li R., Zhao Q.D., Yang Y., Lu Z.H. (2014). Tumor-associated macrophages promote cancer stem cell-like properties via transforming growth factor-beta1-induced epithelial-mesenchymal transition in hepatocellular carcinoma. Cancer Lett..

[66] Bonde A.K., Tischler V., Kumar S., Soltermann A., Schwendener R.A. (2012). Schwendener, Intratumoral macrophages contribute to epithelial-mesenchymal transition in solid tumors. BMC Cancer.

[67] Highfill S.L., Cui Y., Giles A., Smith J.P., Zhang H., Morse E., Kaplan R.N., Mackall C.L. (2014). Disruption of CXCR2-Mediated MDSC Tumor Trafficking Enhances Anti-PD1 Efficacy. Sci. Transl. Med..

[68] Sangaletti S., Tripodo C., Santangelo A., Castioni N., Portararo P., Gulino A., Botti L., Parenza M., Cappetti B., Orlandi R. (2016). Mesenchymal Transition of High-Grade Breast Carcinomas Depends on Extracellular Matrix Control of Myeloid Suppressor Cell Activity. Cell Rep..

[69] Ouzounova M., Lee E., Piranlioglu R., El Andaloussi A., Kolhe R., Demirci M.F., Marasco D., Asm I., Chadli A., Hassan K.A. (2017). Monocytic and granulocytic myeloid derived suppressor cells differentially regulate spatiotemporal tumour plasticity during metastatic cascade. Nat. Commun..

[70] Omland S.H., Wettergren E.E., Mollerup S., Asplund M., Mourier T., Hansen A.J., Gniadecki R. (2017). Cancer associated fibroblasts (CAFs) are activated in cutaneous basal cell carcinoma and in the peritumoural skin. BMC Cancer.

